# Investigation of the effects of physical activity level on functionality level and quality of life in the postpartum period

**DOI:** 10.1186/s41043-023-00368-4

**Published:** 2023-03-30

**Authors:** Halil I. Bulguroglu, Merve Bulguroglu, P. T. Cansu Gevrek

**Affiliations:** 1 Faculty of Health Sciences, Department of Physiotherapy and Rehabilitation, Ankara Medipol University, Ankara, Turkey; 2 Department of Therapy and Rehabilitation, Programme of Physiotherapy, Ankara Medipol University, Vocational School of Health Services, Ankara, Turkey

**Keywords:** Functionality, Physical activity, Postpartum, Quality of life

## Abstract

**Background:**

Physical activity, known to have positive effects in every period of life, may decrease due to anatomical and physiological changes and increased responsibilities in the postpartum period. This study aimed to understand how women's physical activity levels, functional levels, and quality of life are affected in the postpartum period and to emphasize the importance of physical activity levels in the postpartum period.

**Methods:**

The population of our study was planned as a cross-sectional study of postpartum women who applied to a private center. The sample consists of 101 volunteer postpartum women participating in the study. Physical activity levels; with the International Physical Activity Questionnaire (IPAQ), postpartum functional levels; with the Inventory of Functional Status After Childbirth (IFSAC), postpartum quality of life level; with Maternal Postpartum Quality of Life (MAPP-QOL) were evaluated.

**Results:**

It was determined that the amount of physical activity of postpartum women was 928.347 ± 281.27 MET-min/week, which means low physical activity level, and 35.64% were not physically active. The mean total score of IFSAC was 2.13 ± 0.79, and the mean total score of MAPP-QOL was 16.93 ± 6.87. It was determined that there was a positive and significant correlation (*p* < 0.05) between IPAQ and IFSAC (*r* = 0.034) and MAPP-QOL (*r* = 0.214). A significant difference was found when the IFSAC and MAPP-QOL scores were compared between the three groups with different physical activity levels (*p* < 0.05).

**Conclusions:**

As a result, it was observed that the physical activity levels of women in the postpartum period were low, negatively affecting their functionality and quality of life.

## Introduction

The postpartum period is a process that starts after birth and covers the first six months. It is a period in which women experience physiological changes and undertake roles and responsibilities that they have not experienced before. The part of motherhood gained during this period is one of the most challenging roles women experience throughout their lives [1]. At the end of the nine months, the physical changes and discomforts of the mother in transition are added to adapting to the newly acquired roles and responsibilities. Generally, health professionals focus more on the physical health of mothers during this period and give less place to social and emotional needs [2].

The level of functionality is a concept based on realizing the daily vital functions, especially the woman’s basic needs, and the mother must continue her everyday life. The decrease in this level negatively affects the mother's ability to cope with physical changes and the mother-infant harmony, causing a delay in social and emotional recovery [3, 4]. All these affect the mother's quality of life, a multidimensional concept that can affect many aspects of life and cause the postpartum process to be adversely affected [5].

Physical activity, defined as all kinds of body movements performed by contraction of skeletal muscles, requiring energy expenditure above the basal level, has positive effects on human life physiologically and psychologically in every period [6].

Changes in the postpartum period and newborn responsibilities cause a decrease in the physical activity level of the mother. It is known that regular physical activity during this period accelerates the mother's physical recovery and positively affects her mood and quality of life [7].

Physical activity in the postpartum period; improves blood circulation, strengthens abdominal and spinal muscles, stimulates lactation, accelerates uterine recovery, prevents urogynecological dysfunction, and improves mothers' mental and physical condition. All these effects make it easier for mothers to perform their daily activities [8].

In the guidelines published by the American Society of Gynecology and Obstetrics, it is stated that women should do moderate-intensity exercise for at least 150 min, spread over every day of the week, if possible, during the postpartum period [9].

Several studies examine the effects of physical activity on women's quality of life and depression levels in the postpartum period [10, 11]. However, there is no study in the literature evaluating the relationship between the level of functionality of the mother, which is important for mother and child health, and the level of physical activity.

This study aims to understand how the physical activity levels, functional levels, and quality of life of postpartum women affect and to emphasize the importance of interventions that increase physical activity levels by drawing attention to the adverse effects of decreased physical activity, especially in new mothers.

## Methods

### Study design and population

Before the study, the power analysis performed to determine the sample size determined that 100 people were needed for the correlation analysis to be performed by taking the Pearson correlation coefficient *r* = 0.30 with 80% power (alpha = 0.05, bidirectional) in the G*power program. Considering the 20% dropout assumption, 126 postpartum volunteer women who applied to a private center between 22/06/2022 and 22/07/2022 were invited to our cross-sectional study. Twenty of them wanted to be excluded from the study. Five women left the study unwillingly to make the evaluations, and the study was completed with 101 postpartum women. Inclusion criteria were defined as being between 6 weeks and six months postpartum, aged between 20 and 38 years, having given birth for the first time, having a single baby, not having any birth anomaly in oneself or the baby, and accepting to be included in the study. Women with multiple pregnancies and chronic diseases such as hemodynamically significant heart disease, restrictive lung diseases, diabetes, and hypertension were excluded from the study. Before starting the study, ethical approval was obtained from Ankara Medipol University Non-Interventional Clinical Research Ethics Committee (Date: 20/06/2022 Decision No: 0120). The study was conducted by the Helsinki Principles.

### Measuring methods

The individuals included in the study were evaluated with data collection forms filled out through questionnaires. Information was obtained from the individuals' demographic (age, height, weight, body mass index, education level, postpartum week). In addition, physical activity levels, postpartum functional levels, and quality of life were evaluated.

The International Physical Activity Questionnaire (IPAQ) was used to assess physical activity level [12]. The questionnaire, consisting of seven questions covering activities in the last seven days, can be administered by individuals and provides information about the time people spend in moderate to vigorous activities. The Turkish version of IPAQ was used in our study [13]. In the classification of physical activity levels; physically inactive (< 600 MET-min/week), low physical activity level (600–3000 MET-min/week), and sufficient physical activity level (beneficial for health) (> 3000 MET-min/week) are used [14].

Postnatal functional levels of individuals were evaluated with the Inventory of Functional Status After Childbirth (IFSAC) [3]. The IFSAC consists of five subscales, including five dimensions of functional status and 36 four-point Likert-type questions to determine postpartum recovery. These include domestic, social, and community activities, baby care responsibilities, self-care, and professional activities. The total score is calculated by dividing the scores of all answered items by the number of answered items. Each question of the IFSAC has been evaluated over four points (one to four). A high score (close to four) indicates high functional status. The Turkish version of the IFSAC was used in our study [15].

Individuals' postpartum quality of life was evaluated with the Maternal Postpartum Quality of Life (MAPP-QOL) [16]. Postpartum quality of life is a scale that is evaluated according to the perception of the mother and consists of five sub-dimensions and a total of 40 items. Sub-dimensions of the scale; kinship consists of family-friend (nine items), socioeconomic (nine items), spouse (five items), health (eight items), and psychological (nine items) dimensions. The scale assesses how satisfied and important mothers feel at four to six weeks post-discharge postpartum. The scale consists of two parts. In the first part, satisfaction with each item is questioned, and in the second part, the importance is questioned. To calculate the quality of life scale scores; 3.5 is subtracted from each of the satisfaction items from one to six (thus, the figures are -2.5, -1.5, -0.5, 0.5, 1.5, 2.5), and the scores obtained from the satisfaction dimension are multiplied with the same items in the significance dimension of the scale. The scores obtained after the procedure are summed up and divided by the number of scale questions (40 items), and a fixed value (15) is added to the number obtained from the section to avoid negative results, and the result is found. Thus, the Quality of Life Scores is in the range of 0–30. The higher the score obtained from the scale, the higher the quality of life after birth; lower scores indicate low quality of life after birth (15, 16). Our study used the Turkish version of the MAPP-QOL [17].

### Statistical analyses

Statistical analyses of the study were performed using the "Statistical Package for Social Sciences" (SPSS) version 26.0 (SPSS inc., Chicago, IL, USA). Visual (histogram, probability graphs) and analytical methods (Kolomogrov-Smirnov/Shapiro–Wilk's test) were used to define whether the variables were normally distributed or not. Numerical variables with normal distribution are shown as mean ± standard deviation. Pearson correlation analysis determined the relationship between physical activity levels, quality of life, and functionality levels.

One-way ANOVA analysis of variance was used to determine the relationship between the quality of life and functionality levels of the groups with three different physical activity levels.

## Results

101 volunteer postpartum women were included in the study. Age, height, weight, BMI, postpartum weeks, and education levels of the individuals included in the study are given in Table 1.

**Table 1:** Demographics of postpartum women

	Postpartum women (*n* = 101)	
	X ± SD	
Age (years)	26.32 ± 2.98	
Height (cm)	165.72 ± 4.12	
Weight (kg)	64.228 ± 7.84	
BMI (kg/ cm^2^)	24.25 ± 2.17	

	*n*	%
*Education level*		
Primary School	4	3.96
High School	16	15.84
University	73	72.28
Master's Degree	8	7.92
*Postpartum week*		
4th week	8	7.92
5th week	28	27.72
6th week	34	33.67
7th week	31	30.69

X ± SD: mean ± SD, cm: centimeters, kg: kilograms, BMI: body mass index, n:sample size

The average amount of physical activity of postpartum women was found to be 928.347 ± 281.27 MET-min/week (Table 2). The mean total score of IFSAC, in which postnatal functional levels were evaluated, was 2.13 ± 0.79. The mean total score of MAPP-QOL was 16.93 ± 6.87 (Table 2).

**Table 2:** Physical activity, postpartum functional status and postpartum quality of life measurement results of postpartum women

	Postpartum women (*n* = 101)
	X ± SD
IPAQ Total physical activity (MET-min/week)	928.347 ± 281.27
IFSAC Total (1–4)	2.13 ± 0.79
MAPP-QOL Total (0–30)	16.93 ± 6.87

X ± SD: mean ± SD, IPAQ: International Physical Activity Questionnaire, MET: metabolic equivalent, min: minute, IFSAC: Inventory of Functional Status After Childbirth, MAPP-QOL: Maternal Postpartum Quality of Life, n:sample size

When Table 3 is examined, it is seen that 35.64% of postpartum women are not physically active, 50.5% have low physical activity levels, and 13.86% are sufficient to maintain their health.

**Table 3:** Physical activity levels of postpartum women

	Postpartum women (n = 101)	
	*n*	%
Physical activity level		
Physically Inactive (< 600 MET- min/week)	36	35.64
Low Physical Activity Level (600 – 3000 MET-min/week)	51	50.5
Physical Activity Level Sufficient (> 3000 MET-min/week)	14	13.86

MET: metabolic equivalent, min: minute, n: sample size

Table 4 shows the correlation coefficients between women's IPAQ mean scores and the total mean scores of IFSAC and MAPP-QOL. It was determined that there was a moderate (*r* = 0.034) positive correlation between IPAQ and IFSAC (*p* < 0.05), and a weak (*r* = 0.214) positive correlation between IPAQ and MAPP-QOL (*p* < 0.05).

**Table 4:** The relationship between average amount of physical activity of postpartum women and postpartum functional status and postpartum quality of life total scores

	Postpartum women (*n* = 101)
	IPAQ (MET-min/week)
IFSAC Total	r: 0.034 p: 0.031*
MAPP-QOL Total	r: 0.214 p: 0.027*

IPAQ: International Physical Activity Questionnaire, MET: metabolic equivalent, min: minute, IFSAC: Inventory of Functional Status After Childbirth, MAPP-QOL: Maternal Postpartum Quality of Life

*p < 0.05

Table 5 compares the mean scores of IFSAC and MAPP-QOL according to the level of physical activity. When IFSAC and MAPP-QOL scores were compared according to physical activity levels, a significant difference was found between the three groups according to physical activity levels (*p* < 0.05, Table 5).

**Table 5:** Investigation of the relationship between postpartum functional status and postpartum quality of life total scores according to physical activity levels of postpartum women

	Physical activity level			
	Physically Inactive (< 600 MET- min/week)	Low Physical Activity Level (600–3000 MET-min/week)	Physical Activity Level Sufficient (> 3000 MET-min/week)	Test and p-value
IFSAC Total	2.01 ± 0.93	2.38 ± 1.01	3.20 ± 0.37	F = 9.467 **p = 0.035**
MAPP-QOL Total	12.43 ± 6.44	15.93 ± 5.97	20.15 ± 4.31	F = 8.328 **p = 0.011**

Bold values indicate* p* < 0.05

MET: metabolic equivalent, min: minute, IFSAC: Inventory of Functional Status After Childbirth, MAPP-QOL: Maternal Postpartum Quality of Life

## Discussion

This study showed that women's physical activity levels during the postpartum period were low, and their functional and quality of life levels were adversely affected.

Our study found that the physical activity levels of postpartum women were low, especially 35.64% of physically inactive women, and the mean IPAQ scores were 928.347 ± 281.27.

Similar to our study, studies show that the physical activity levels of postpartum women are insufficient [10, 18]. However, studies are showing that lack of physical activity in the postpartum period leads to decreased weight loss [19], increased pain [20], and deterioration in sleep quality [21], especially depression [10, 11]. The results of these studies show us the negativities of inactive life and emphasize the importance of physical activity during motherhood, which is one of the critical processes of life. In our study, similar to other studies, the functional and quality of life levels of women with low physical activity levels and who are not physically active were found to be low.

In our study, the mean total score of MAPP-QOL was 16.93 ± 6.87. In addition, a positive weak correlation was found between IPAQ and MAPP-QOL, and it was observed that the mean quality of life score of postpartum women with high physical activity levels was higher than other levels. We think that the reason for the low correlation is due to the low number of participants.

There are studies in the literature that have similar results to our study. Bahadoran et al., in their study with 91 pregnant women, stated that an increase in the level of physical activity increases the well-being of women [22].

In another study, it was stated that the quality of life of women in the postpartum period is low, and this low level may cause various problems in women [23].

Another study determined that the quality of life levels of women with insufficient physical activity levels were lower than those of women with higher physical activity levels [10].

We think the increase in physical activities during the postpartum period will make women feel more energetic, strong, and fit and will positively affect many parameters, such as motherhood roles and quality of life.

Although a few studies in the literature emphasize the importance of the functional level of the mother in the postpartum period [1, 2], no study evaluating its relationship with physical activity has been found. As a result of our study, the mean IFSAC total score was 2.13 ± 0.79. In addition, a positive moderate correlation was found between IPAQ and IFSAC, and it was observed that the functional levels of postpartum women decreased with the decrease in physical activity.

The level of functionality, which means the mother's readiness to undertake self-care, social, social, and professional activities, and baby care, gradually decreases after birth.

In their study, Sanli and Oncel pointed out that it takes longer than six months for the mother to reach her pre-pregnancy functional level and stated the importance of providing the necessary support to mothers quickly adapt to the postpartum period [2].

Fathi et al. stated that the increase in depression levels negatively affected the functional levels of women and reported that treatments for depression would increase the functional levels of women [1].

It is known that physical activity has positive effects on depression in the whole population. In addition, the decrease in the physical activity levels of postpartum women and the process they are in may decrease the satisfaction levels they receive from life, and their functional levels may be negatively affected. These results may affect the motivational aspects of women in the postpartum period and their motherhood roles. Increasing the physical activity levels of postpartum women provides both the physiological benefits of physical activity in women and the motivational contribution provided by activity and mental relaxation. This will positively affect the mother's care and social and professional functions, especially mother-infant development.

The fact that the questionnaires used in our study to evaluate both qualities of life and functional level are specific to the postpartum period is one of the strengths of our study. In addition, our study is the first study investigating the effect of physical activity level on functional status in postpartum women. The most important limitation of our study was not questioning the types of physical activity performed by postpartum women.

## Conclusions

Our study will guide the literature that with the decrease in women's physical activity levels during the postpartum period, their quality of life and functional levels decrease and that directing postpartum women to physical activities is at least as necessary as other roles. In future studies, examining the effects of different types of physical activity on various factors in postpartum women is necessary.

## Data Availability

Data of participants who agreed to the public distribution of data are available from the corresponding author upon reasonable request.

## Abbreviations

Cm: Centimeters
Kg: Kilograms
BMI: Body mass index
IPAQ: International Physical Activity Questionnaire
MET: Metabolic equivalent
Min: Minute
IFSAC: Inventory of Functional Status After Childbirth
MAPP-QOL: Maternal Postpartum Quality of Life002E

## References

[1] Fathi F, Mohammad-Alizadeh-Charandabi S, Mirghafourvand M (2018). Maternal self-efficacy, postpartum depression, and their relationship with functional status in Iranian mothers. Women Health.

[2] Sanli Y, Oncel S (2014). Evaluation of the functional status of woman after childbirth and effective factors. J Turk Soc Obstet Gynecol.

[3] Fawcett J, Tulman L, Myers ST (1988). Development of the inventory of functional status after childbirth. J Nurse Midwifery.

[4] Doering Runquist JJ, Morin K, Stetzer FC (2009). Severe fatigue and depressive symptoms in lower-income urban postpartum women. West J Nurs Res.

[5] Edhborg M, Seimyr L, Lundh W, Widstrom AM (2000). Fussy child-difficult parenthood? Comparisons between families with a “depressed” mother and non-depressed mother 2 month postpartum. J Reprod Infant Psychol.

[6] Mikkelsen K, Stojanovska L, Polenakovic M, Bosevski M, Apostolopoulos V (2017). Exercise and mental health. Maturitas.

[7] Blum JW, Beaudoin CM, Caton-Lemos L (2004). Physical activity patterns and maternal well-being in postpartum women. Matern Child Health.

[8] Evenson KR, Mottola MF, Owe KM, Rousham EK, Brown WJ (2014). Summary of international guidelines for physical activity following pregnancy. Obstet Gynecol Surv.

[9] American College of Obstetricians and Gynecologists Committee Opinion (2020). Physical activity and exercise during pregnancy and the postpartum period. Obstet Gynecol.

[10] Okyay EK, Ucar T (2018). The effect of physical activity level at postpartum period on quality of life and depression level. Med Sci.

[11] Kołomańska-Bogucka D, Mazur-Bialy AI (2019). Physical activity and the occurrence of postnatal depression—a systematic review. Medicina.

[12] Craig CL, Marshall AL, Sjöström M, Bauman AE, Booth ML, Ainsworth BE, Pratt M, Ekelund U, Yngve A, Sallis JF, Oja P (2003). International physical activity questionnaire: 12-country reliability and validity. Med Sci Sports Exerc.

[13] Saglam M, Arikan H, Savci S, Inal-Ince D, Bosnak-Guclu M, Karabulut E, Tokgozoglu L (2010). International physical activity questionnaire: reliability and validity of the turkish version. Percept Mot Skills.

[14] Hagströmer M, Oja P, Sjöström M (2006). The International Physical Activity Questionnaire (IPAQ): a study of concurrent and construct validity. Public Health Nutr.

[15] Ozkan S, Sevil U (2007). The study of validity and reliability of inventory of functional status after childbirth. TAF Prev Med Bull.

[16] Hill PD, Aldag JC, Hekel B, Riner G, Bloomfield P (2006). Maternal postpartum quality of life questionnaire. J Nurs Meas.

[17] Altuntug K, Ege E (2012). The validity and reliability of the Turkish version of the postpartum quality of life scale. J Anatolia Nurs Health Sci.

[18] Adeniyi AF, Ogwumike OO, Bamikefa TR (2013). Postpartum exercise among nigerian women: ıssues relating to exercise performance and self-efficacy. ISRN Obstet Gynecol.

[19] Ha AVV, Zhao Y, Binns CW, Pham NM, Nguyen P, Nguyen CL, Chu TK, Lee AH (2020). Postpartum physical activity and weight retention within one year: a prospective cohort study in Vietnam. Int J Environ Res Public Health.

[20] Girard MP, O'Shaughnessy J, Doucet C, Ruchat SM, Descarreaux M (2020). Association between physical activity, weight loss, anxiety, and lumbopelvic pain in postpartum women. J Manipulative Physiol Ther.

[21] Bay H, Eksioglu A, Sogukpinar N, Turfan CE (2022). The effect of postpartum sleep quality on mothers’ breastfeeding self-efficacy level. Early Child Dev Care.

[22] Bahadoran P, Tirkesh F, Oreizi HR (2014). Association between physical activity 3–12 months after delivery and postpartum well-being. Iran J Nurs Midwifery Res.

[23] Ercel O, Sut HK (2020). Sleep quality and quality of life in postpartum woman. J Turk Sleep Med.

